# Antioxidant Capacity, Cytotoxicity and Antimycobacterial Activity of Madeira Archipelago Endemic *Helichrysum* Dietary and Medicinal Plants

**DOI:** 10.3390/antiox3040713

**Published:** 2014-10-31

**Authors:** Sandra C. Gouveia-Figueira, Carla A. Gouveia, Maria J. Carvalho, Ana I. Rodrigues, Malin L. Nording, Paula C. Castilho

**Affiliations:** 1 Centro de Química da Madeira, CCCEE, Universidade da Madeira, Campus Universitário da Penteada, piso 0, Funchal 9000-390, Portugal; E-Mails: carlaapgouveia@gmail.com (C.A.G.); mariajmcarvalho@hotmail.com (M.J.C.); castilho@uma.pt (P.C.C.); 2 Department of Chemistry, Umeå University, Umeå SE-901 87, Sweden; E-Mail: malin.nording@chem.umu.se; 3 Department of Pharmacology and Clinical Neuroscience, Umeå University, Umeå SE-901 87, Sweden; 4 Unidade de Geologia Marinha, Laboratório Nacional de Energia e Geologia, I.P., Estrada da Portela, Zambujal—Alfragide Apartado 7586, Amadoram 2720-866, Portugal; E-Mail: ana.rodrigues@lneg.pt

**Keywords:** antioxidant, antimycobacterial, *Artemia salina*, *Helichrysum*, chemometrics

## Abstract

The potential bioactivity of dietary and medicinal endemic *Helichrysum* plants from Madeira Archipelago was explored, for the first time, in order to supply new information for the general consumer. *In vitro* antioxidant properties were investigated using DPPH, ABTS^•+^, FRAP and β-Carotene assays, and the total phenolic content (TPC) and total flavonoid content (TFC) were also determined. Although the results generally showed a large variation among the three analyzed plants, the methanolic extracts showed the highest antioxidant capacity. Exception is made for *H. devium* *n-*hexane extract that showed good radical scavenger capacity associated to compounds with good reducing properties. In the *Artemia salina* toxicity assay and antimycobaterial activity, *H. devium* was the most potent plant with the lowest LD_50_ at 216.7 ± 10.4 and MIC ≤ 50 μg·mL^−1^. Chemometric evaluation (Principal Component Analysis—PCA) showed close interdependence between the ABTS, TPC and TFC methods and allowed to group *H. devium* samples.

## 1. Introduction

There is a growing effort in the search for natural compounds displaying biological activities associated with low cytotoxicity.

High levels of free radicals and reactive oxygen species (ROS) such as superoxide anion (O_2_^•−^), hydroxyl radical (OH^•^) and peroxyl radical (ROO^•^) produced *in vivo* are extremely reactive. Those species have been associated with severe pathological processes such as cancer, atherosclerosis, neurodegenerative and cardiovascular diseases, inflammation and aging, as well as to food deterioration [1,2].

Edible plants have been described in traditional medicines to treat several diseases and conditions. Many of these plants have an important antioxidant effect without some of the disadvantages presented by the synthetic antioxidants [3].

A large number of antioxidants isolated from higher plants are secondary metabolites, such as phenolic compounds [4].

Tuberculosis is caused by *Mycobacterium tuberculosis* (MBT) and, to a low level, by *Mycobacterium bovis* and *Mycobacterium* *africanum.* Tuberculosis is a leading cause of mortality worldwide, infecting about nine million people and killing about two million people annually [5]. The new infections and reactivation of latent tuberculosis is rising mainly in individuals with compromised immune systems, such as cases of HIV-positive individuals [6].

Natural purified compounds and extracts from plants, microorganisms and marine organisms with high antioxidant capacity have been described as inhibiting *Mycobacterium tuberculosis* (MBT). In the last decades, several literature reviews have been reported regarding natural compounds active against MBT [6,7,8,9,10,11,12].

Plants of the genus *Helichrysum* Mill. belong to the Asteraceae family and comprise more than 500 species [13]. They are normally used as herbal infusions and are associated with numerous biological activities such as antioxidant, antimicrobial, anti-inflammatory, anti-allergic, in addition to relief of abdominal pain, heart burn, cough, cold and wounds [14,15].

In Madeira Archipelago (Portugal), there are four endemic species of *Helichrysum* and three (*Helichrysum devium*, *Helichrysum melaleucum* and *Helichrysum obconicum*) are reported as being largely used in the local traditional medicine and diet [16].

*Helichrysum devium* Johns. and *Helichrysum melaleucum* Rchb. ex. Holl. are used against respiratory diseases, such as bronchitis and pharyngitis and also as a cough relief. *Helichrysum obconicum* DC. is used in infusions as a digestive, to relieve stomachic pain, as well as for intestinal diseases [16].

In our previous work [13,17,18], characterization and quantification of the phenolic compounds of these three species by HPLC-DAD-ESI/MS^n^ was reported. Phenolic compounds, namely flavonoids and hydroxycinnamic acids, were found to be the major components.

To the best of our knowledge, this paper is the first study of the antioxidant capacity (DPPH, ABTS^•+^, FRAP and β-Carotene), cytotoxicity and antimicrobial activity of these three *Helichrysum* dietary medicinal plants.

The interrelations between these parameters were studied using chemometric methods (PCA analysis) for data evaluation.

## 2. Materials and Methods

### 2.1. Chemicals

The following reagents were purchased from Merck (Darmstadt, Germany): potassium persulfate (99%), sodium chloride (99.5%), disodium phosphate dodecahydrated (99%), glacial acetic acid (100%), sodium carbonate (p.a.), and ferrous sulfate heptahydrate (99%), from Fluka (Lisbon, Portugal), 2,2-diphenyl-1-picrylhydrazyl (DPPH) (>95%), Trolox (≥99.8%), 2,2′-azinobis-(3-ethylbenzthiazoline-6-sulfonic acid) (ABTS) (≥99%), 2,4,6-tri(2-pyridyl)-*s*-triazine (TPTZ) (≥99.0%), β-Carotene (≥97%, UV), Tween 40 and Folin–Ciocalteu’s phenol reagent were purchased from Fluka (Lisbon, Portugal). Potassium chloride (>99.5%), gallic acid (99%), rutin (≥98%, HPLC) and ferric chloride hexahydrated (97%–102%) were purchased from Panreac (Barcelona, Spain); potassium dihydrogen phosphate (99.5%), aluminum chloride (98%), sodium acetate trihydrate (pure) and dimethylsulfoxide (97%) were purchased from Riedel-de Haën (Hanover, Germany). Alamar Blue solution was purchased from Trek Diagnostics (Westlake, OH, USA).

All solvents for plant extraction were AR grade, from Fisher (Lisbon, Portugal). Water was purified by a Milli-Q Gradient system (Millipore, Milford, MA, USA).

### 2.2. Instruments

Spectrophotometric measurements were performed on a Perkin Elmer UV-Vis spectrometer Lambda 2 (Perkin Elmer, Ueberlingen, Germany) equipped with a water thermostatic cell holder with glass cells of 1 cm optical path. The UV-Vis measurements in the β-Carotene method were performed using a model Victor3 microtiter reader (Perkin–Elmer, Ueberlingen, Germany).

Fluorescence was measured in a plate fluorometer (Fluoroskan Ascent FL, Thermo, Finland) at an excitation wavelength of 490 nm and an emission wavelength of 540 nm, and relative fluorescence units (*rfu*) were recorded.

### 2.3. Plant Material

Specimens of three endemic species of *Helichrysum* were collected during May and June from the northern coast of Madeira Island. They were identified by taxonomist Fátima Rocha and vouchers were deposited in the Madeira Botanical Garden Herbarium collection. The total aerial parts were dried at room temperature (protected from direct sunlight) and ground into a fine powder by a mechanical grinder.

Each sample (100 g·plant·L^−1^ of solvent) was extracted through sequential maceration with four organic solvents of increasing polarity (*n*-hexane, chloroform, ethyl acetate and methanol), at room temperature for 24 h. The extracts were filtered and evaporated to dryness under reduced pressure in a rotary evaporator, at 40 °C.

For the *Mycobacterium tuberculosis* studies, crude methanolic extracts were obtained by plant maceration for 48 h followed by filtration and concentration to dryness.

### 2.4. Antimycobacterial Activity

#### 2.4.1. *Mycobacterium* Strains

The following *Mycobacterium* species was obtained from the American Type Culture Collection (ATCC): *Mycobacterium tuberculosis* H37Rv (27294).

#### 2.4.2. Inoculum Preparation for Biological Assays

The strain was cultured at 37 °C in Middlebrook 7H9 broth (7H9), supplemented with 0.2% glycerol and 10% OADC enrichment (oleic acid, albumin, dextrose, catalase; Difco) until *log* phase growth was achieved. The inocula for microcolorimetric assay was prepared by diluting *log* phase growth cultures with sterile 7H9 to the McFarland No. 1 turbidity standard, and were then further diluted 1:20 in 7H9. The working suspension was prepared just prior to inoculation of the microplate.

#### 2.4.3. Antimycobacterial Screening by Microplate Alamar Blue Assay

The methodology used was based on that described by Jimenez-Arellanes *et al.* [19].

Stock solutions of each sample were prepared in DMSO at a concentration of 20 g·L^−1^. Serial dilutions of each sample were prepared; final concentrations ranged from 50 to 200 mg·L^−1^. Duplicates of each sample were made per plate and each experiment was repeated at least twice. Bacterial suspension (100 μL) was added to test wells and to controls. A 1:10 diluted control was included in each plate, representing the growth of 10% of the bacterial population tested (10% control). The plates were incubated at 37 °C for 5 days and after that they were developed by adding 20 μL of Alamar Blue solution (Trek Diagnostics, Westlake, OH, USA) to each well. The plates were reincubated, at 37 °C, for 24 h. Fluorescence was measured in a plate fluorometer (Fluoroskan Ascent FL, Thermo, Finland) at an excitation wavelength of 490 nm and an emission wavelength of 540 nm, and relative fluorescence units (*rfu*) were recorded. Wells with a well-defined pink color were scored as positive for growth.

The minimal inhibitory concentration (MIC) was defined as the lowest sample concentrations that prevent a color change to pink and that presented *rfu* values lower than those presented by the 10% growth control.

Extracts were considered active if they gave a MIC < 200 μg·mL^−1^. Extracts active at concentrations lower than 50 μg·mL^−1^ were further tested at lower concentrations (down to 6.25 μg·mL^−1^).

### 2.5. Artemia Salina Toxicity Evaluation

The brine shrimp toxicity assay was adapted from the method reported by Dey and Harborne [20].

Filtered seawater was added to a small tank and air was bubbled through it, using an aquarium pump; shrimp eggs were added to the aired water and left to incubate at room temperature for 48 h; fully developed shrimp larvae were collected at this point and immediately used.

For each sample, stock solutions were prepared in DMSO with a concentration (m/v) of 20 mg·mL^−1^. The tested solutions presented the following concentrations: 5 mg·mL^−1^; 1 mg·mL^−1^; 0.5 mg·mL^−1^ and 0.1 mg·mL^−1^. 5 mL of seawater, 50 μL of each sample and 10 shrimp larvae (30 per dilution) were added to each vial. The percentage of larvae deaths was calculated after 24 h of incubation. The LD_50_ was calculated using a linear regression equation. When high mortality was observed, intermediary concentrations were tested (3.5 and 2.5 mg·mL^−1^). For each experiment a control was performed consisting of adding 50 μL of DMSO.

### 2.6. Determination of Total Phenolic Content (TPC)

Total phenolic contents were determined by the Folin–Ciocalteu method described by Zheng and Wang [21] with some modifications. Plant extracts were dissolved in methanol to yield a final concentration (w/v) of 10 mg·mL^−1^. Of each diluted extract, 50 μL was mixed with 1.25 mL of Folin–Ciocalteau reagent (diluted 1:10 fold) and 1 mL of 7.5% sodium carbonate solution. The mixture was incubated for 30 min, at room temperature, and then absorbance was measured at 765 nm. The TPC was expressed as gallic acid equivalents per 100 g of dried plant (mg GAE 100 g^−1^).

### 2.7. Determination of Total Flavonoid Content (TFC)

Total flavonoid content was measured using a colorimetric aluminum chloride method [22]. Ten mg of each extract was dissolved in 5 mL of methanol and 0.5 mL of this diluted extract was mixed with 1.5 mL of methanol, 2.8 mL of water, 0.1 mL of potassium acetate (1 M) and 0.1 mL of aluminum chloride (10% in methanol). After incubation for 30 min, the decrease in absorbance was measured at 415 nm. The TFC was calculated using a standard calibration curve of rutin solutions (12.5 to 100 μg·mL^−1^), and expressed as milligrams of rutin equivalents per 100 g of dried sample (mg RUE 100 g^−1^).

### 2.8. Antioxidant Assays

#### 2.8.1. 2,2-Diphenyl-1-Picrylhydrazyl (DPPH) Radical Scavenging Activity

The DPPH assay was executed according to [23] with minor modifications. One hundred μL of extract solution (10 mg·mL^−1^) was added to 3.5 mL of a 0.06 mM methanol DPPH radical solution. The decrease in absorbance at 516 nm was measured every minute during a 30 min period, in the dark. The DPPH radical scavenging activity was expressed based on the Trolox calibration curve as μmol Trolox equivalent per 100 g of dried plant (μmol eq. Trolox 100 g^−1^ dried plant).

#### 2.8.2. 2,2′-Azinobis-(3-Ethylbenzthiazoline-6-Sulfonic Acid) (ABTS^•+^) Radical Scavenging Activity

A modified method, initially reported by [24] was used for the determination of the antioxidant activity by the method of decolorization of free radical ABTS^•+^. The ABTS**^•^**^+^ radical was prepared by reacting 50 mL of 2 mM ABTS^•+^ aqueous solution with 200 μL of 70 mM K_2_S_2_O_8_ solution. This mixture was kept in the dark for 16 h, at room temperature. Before each experiment, the ABTS**^•^**^+^ solution was diluted with pH 7.4 phosphate buffered saline (PBS) solution to an initial absorbance of 0.700 ± 0.021 at 734 nm.

Briefly, an aliquot of 100 μL of extract solution (10 mg·mL^−1^) was added to 1.8 mL of ABTS^•+^ solution and the absorbance at 734 nm was recorded during 6 min. Results were expressed in terms of μmol Trolox equivalent per 100 g of dried plant antioxidant capacity (μmol eq. Trolox 100 g^−1^ dried plant).

#### 2.8.3. Ferric Reducing Activity (FRAP Assay)

The ferric reducing activity of the plant extracts was estimated based on the FRAP assay [25]. At low pH, the ferric-tripyridyltriazine (Fe^3+^-TPTZ) orange complex is reduced to a blue-colored ferrous complex (Fe^2+^-TPTZ) by the action of electron-donating antioxidants. This reaction was followed with an increase in absorbance at 593 nm. FRAP reagent was prepared daily by mixing 2.5 mL of solution FeCl_3_·6H_2_O (20 mM), 2.5 mL of solution TPTZ (10 mM in 40 mM of HCl) and 25 mL of acetate buffer 0.3 M (pH 3.6) and was incubated at 37 °C. For each analysis, 30 μL of solution (1 mg·mL^−1^) was added to 180 μL of distilled water and 1.8 mL of FRAP solution. The variance absorbance of the reaction mixture was recorded at 593 nm in 15 s intervals, during 30 min against methanol as blank. Methanolic solutions of known Fe (II) concentration, in the range of 2.00–500 mM (FeSO_4_·7H_2_O) were used to prepare the calibration curve. The FRAP results were expressed as mmol FeSO_4_·7H_2_O per mg of dried plant (mmol Fe(II) mg^−1^).

#### 2.8.4. β-Carotene (BC) Bleaching Assay

This method was based on that described by Siddhuraju and Becker [26]. Briefly, 2 mL of β-Carotene solution 0.2 mg·mL^−1^ in chloroform was added to a round-bottom flask (250 mL) containing 0.04 mL of linoleic acid and 200 mg of Tween 40. The chloroform was completely evaporated using nitrogen and then 50 mL of oxygenated ultra-pure water, obtained by bubbling air through the water for 15 min was added, and the mixture was vigorously shaken. The resulting emulsion was freshly prepared before each experiment. Stock solutions of the extracts were prepared in ethanol (1 mg·mL^−1^). An aliquot of 250 μL of the β-Carotene/linoleic acid emulsion was distributed in each of the wells of the 96-well microtiter plates and 30 μL of the sample solutions were added. An equal amount of ethanol was used for the control sample. The samples were then subjected to thermal autoxidation at 45 °C for 210 min. The absorbance of the solution was measured at 490 nm by taking measurements at 15 min intervals. The antioxidant activity (AA) of each sample was evaluated in terms of the bleaching of β-Carotene using the following equation: AA (%) = (1 − (A_0_ − A_t_/A’_0_ − A’_t_)) × 100, where A_0_ and A’_0_ are the absorbance values measured at zero incubation time for the test and control respectively and A_t_ and A’_t_ are the corresponding absorbance values measured after incubation for 210 min. All samples were assayed in triplicate.

### 2.9. Statistical Analysis

All measurements were performed in triplicate and results are expressed as mean ± SD.

Significant differences in antioxidant activity and total phenolic content of the different extracts were determined using one-way ANOVA. The Pearson correlation coefficients were determined using SPSS. The statistical probability was considered to be significantly different at the level of *p* < 0.05.

Principal components analysis (PCA) was done on the results of TPC, TFC, DPPH, ABTS, FRAP, BC and *Artemia salina* of the total aerial parts extracts of *Helichrysum* plants using SIMCA software v.13 (Umetrics AB, Umeå, Sweden).

## 3. Results and Discussion

### 3.1. Extraction Yield

The percentage yields of all analyzed extracts of *Helichrysum* species are presented in Table 1. Yields ranged from 0.18% (*H. melaleucum*, ethyl acetate) to 15.0% (*H. obconicum*, methanol), and extraction with methanol was the most efficient presenting a higher yield.

### 3.2. Antimycobacterial Activity

**Figure 1:**
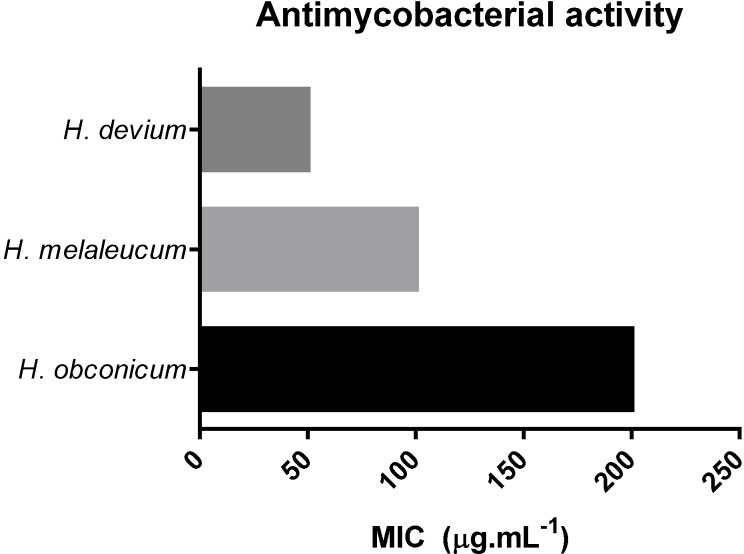
Antimycobacterial activity of methanolic crude extracts of *Helichrysum* plants.

Given the traditional uses of *Helichrysum* plants in Madeira against respiratory problems such as bronchitis and pharyngitis, preliminary studies over *Mycobacterium tuberculosis* were performed. The methanolic crude extracts of *H. devium*, *H. melaleucum* and *H. obconicum* were evaluated for their activity against *M. tuberculosis* H37Rv and the results are shown in Figure 1. *H. devium* was very potent against *M. tuberculosis* with a MIC equal to 50 μg·mL^−1^ and *H. melaleucum* gave a moderate activity with 100 μg·mL^−1^, while *H. obconicum* presented low activity, with a MIC of 200 μg·mL^−1^.

Others have studied the antimycobacterial activity of other *Helichrysum* species. *Helichrysum melanacme* ethanolic extract showed a MIC of 500 μg·mL^−1^ against *M. tuberculosis* H37Rv. Biofractionation of this extract led to isolation of two chlacones with MIC of 50 μg·mL^−1^ [27].

**Table 1:** Experimental determinations of total phenolic (TPC), flavonoid content (TFC), antioxidant capacity (DPPH, ABTS, FRAP and β-Carotene) and toxicity activity (mean ± SD) from total aerial parts of *Helichrysum* species extracts.

Plant Extracts		TPC (mg GAE 100 g^−1^)	TFC (mg RUE 100 g^−1^)	DPPH	ABTS	FRAP mmol Fe(II) mg^−1^	BC (%)	Toxicity Activity LD_50_ (μg mL^−1^)	Extraction Yield (%)
				μmol eq. Trolox 100 g^−1^					
*H. devium*	*n*-Hexane	221.4 ± 1.72 ^c^	44.95 ± 0.193 ^d^	289.7 ± 1.03 ^e^	116.4 ± 0.434 ^f^	694.9 ± 3.89 ^b^	62.82 ± 1.58 ^f^	2.36 ^d^	216.7 ± 10.4
	Chloroform	234.6 ± 3.61 ^d^	40.27 ± 0.429 ^c^	234.8 ± 1.09 ^d^	84.57 ± 0.0700 ^d^	852.4 ± 11.1 ^e^	60.62 ± 2.25 ^f^	3.63 ^e^	5025 ± 63.2
	Ethyl acetate	312.7 ± 1.73 ^f^	180.3 ± 10.6 ^f^	236.4 ± 4.41 ^d^	86.12 ± 0.0707 ^d^	2140.0 ± 14.6 ^g^	52.34 ± 1.53 ^d^	3.61 ^e^	*
	Methanol	399.6 ± 1.52 ^g^	302.8 ± 1.19 ^g^	235.4 ± 1.63 ^d^	107.1 ± 0.0706 ^e^	2431.8 ± 12.9 ^h^	46.82 ± 1.75 ^c^	4.85 ^f^	479.0 ± 8.20
*H. melaleucum*	*n*-Hexane	258.9 ± 0.631 ^e^	46.26 ± 0.477 ^d^	137.6 ± 2.66 ^c^	56.94 ± 0.0658 ^b^	354.8 ± 1.35 ^a^	61.02 ± 1.45 ^f^	1.14 ^c^	322.9 ± 11.9
	Chloroform	106.5 ± 0.373 ^b^	56.61 ± 0.663 ^e^	119.2 ± 0.691 ^b^	71.97 ± 0.0348 ^c^	831.0 ± 3.09 ^d^	56.06 ± 1.29 ^e^	1.35 ^c^	752.0 ± 36.6
	Ethyl acetate	NA	NA	NA	NA	NA	NA	0.18 ^a^	3349 ± 102.3
	Methanol	1214 ± 2.11 ^i^	816.9 ± 1.96 ^i^	812.9 ± 1.65 ^g^	286.9 ± 1.28 ^g^	1581.7 ± 7.71 ^f^	22.85 ± 0.29 ^a^	7.64 ^g^	*
*H. obconicum*	Hexane	39.75 ± 0.214 ^a^	19.20 ± 0.251 ^a^	NI	NI	NI	NI	1.23 ^c^	934.5 ± 56.2
	Chloroform	NA	NA	NA	NA	NA	NA	NA	NA
	Ethyl acetate	42.05 ± 0.134 ^a^	23.61 ± 0.195 ^b^	23.78 ± 0.754 ^a^	5.780 ± 0.031 ^a^	741.08 ± 19.9 ^c^	36.43 ± 0.58 ^b^	0.57 ^b^	2,410 ± 88.3
	Methanol	773.4 ± 14.3 ^h^	703.03 ± 2.98 ^h^	638.8 ± 1.50 ^f^	687.9 ± 7.25 ^h^	19,918.3 ± 75.4 ^i^	37.96 ± 0.59 ^b^	15.0 ^h^	1075 ± 71.5

* Values represented as mean ± SD (*n* = 3); Means not sharing the same letter in the same column are significantly different at *p* < 0.05 probability level; NA—Not Analyzed; NI—No Inhibition.

### 3.3. Artemia Salina Toxicity

Naturally occurring compounds can show the therapeutic effect by exhibiting toxicity to the pernicious cells or materials. Regarding the safety of the *Helichrysum* medicinal and dietary plants, evaluation of toxicity was performed *in vitro* with the *Artemia salina* toxicity assay.

The results were expressed as LD_50_ values (concentration able to kill 50% of the brine shrimp larvae). These values were calculated by linear regression using the least squares method. Samples with values of LD_50_ higher than 1,000 μg·mL^−1^ were considered non-toxic [28].

The LD_50_ results obtained for *H. devium*, *melaleucum* and *obconicum* extracts are shown in Table 1.

The *n*-hexane extracts of *H. devium* and *H. melaleucum* presented toxicity on *Artemia salina* with values of 216.7 ± 10.4 and 322.9 ± 11.9 μg·mL^−1^, respectively. The methanolic extract of *H. devium* gave a low LD_50_ value at 479.0 ± 8.20 μg·mL^−1^, suggesting a moderate toxicity capacity.

*H. obconicum* extracts were considered almost non-toxic, the *n*-hexane extract is on the limit with a LD_50_ value of 934.5 ± 56.2 μg·mL^−1^.

Samples rich in flavonoids are known to possess low LD_50_ values against *Artemia salina* [29,30]. Several flavonoids were identified in *H. devium* [13] and *H. melaleucum* [17], while *H. obconicum* was rich in quinic acid derivatives [18]. These differences in the composition can contribute to the different toxicity of each plant.

The higher toxicity of the *n*-hexane extracts can be attributed to the presence of non-polar compounds, such as terpenes, also known for being toxic to *Artemia salina* larvae [28], or other non-identified hexane soluble substances.

### 3.4. Total Phenolic Content (TPC) and Total Flavonoid Content (TFC)

Plant secondary metabolites (phenolics and/or polyphenolics compounds) are known for their antioxidant activity. In this study, therefore, endemic *Helichrysum* species from Madeira Archipelago were evaluated for TPC and TFC (Table 1).

Results showed that TPC and TFC varied considerably for each plant. TFC was 10 times higher for the methanolic extract and *H. melaleucum* gave the biggest amount followed by *H. obconicum* and *H. devium*.

TPC values ranged from 39.75 ± 0.214 to 1214 ± 2.11 mg GAE 100 g^−1^ found in *H. obconicum* *n*-hexanic and *H. melaleucum* methanolic extracts, respectively. For all three species, methanolic extracts showed the highest TPC, and the *n-*hexane extracts of *H. devium* and *H. obconicum* presented the lowest, while for *H. melaleucum* the lowest TPC was for chloroform extract. The TFC varied from 19.20 ± 0.251 to 703.03 ± 2.98 mg RUE 100 g^−1^ (Table 1).

Albayrak *et al.* [31] reported the composition and antioxidant activity of 16 *Helichrysum* species from Turkey. The TPC values (66–160 mg·GAE·g^−1^) were higher than the values that we found for Madeira *Helichrysum* species. However, they also detected different TPC values for different species mainly associated to plant origin.

An exploratory study with *Helichrysum graveolens* [32] showed that ethyl acetate extracts have higher TPC and antioxidant capacity when compared with hexane or methanol extracts. Apigenin was the major component of the most active fraction.

Phenolic compounds are polar, thus normally extracted from plants by polar solvents. The observed high value of phenolic content in non-polar solvents, such as *n*-hexane extracts, is not expected but can be tentatively explained. As stated by Huang *et al.* [33], the Folin–Ciocalteu reagent can also react with other reducing compounds rather than phenolic compounds, such as sugars, amino acids or ascorbic acid, and the final results obtained by this method take into account more than the contribution of the phenolic compounds of the sample. Hence, special attention must be given to the results obtained by this method and whenever possible compare them with HPLC quantification of each individual compound and/or other spectrophotometric methods that can give more information to the type of compounds present in the extract.

### 3.5. Antioxidant Assays

The antioxidant capacity of a complex matrix, such as a plant extract, is attributed to the presence of several components from different classes of compounds. The determination of each individual component contribution to the total antioxidant activity is very demanding and time consuming. Therefore, there are several methods reported for the determination of the whole extract antioxidant capacity. These methods are based on two main mechanisms: single electron transfer (ET) and hydrogen atom transfer (HAT). In HAT assays, the antioxidant and the sample compete for the generated peroxyl radicals. ET-based assays measure the capacity of an antioxidant to reduce an oxidant which changes color when reduced. DPPH, ABTS and FRAP belong to the ET methods [33].

A special remark must be made regarding the comparison of antioxidant assays results with those published in the literature since small variations on the experimental conditions can affect, to a large extent, the results obtained.

#### 3.5.1. DPPH^•^ Radical Scavenging Activity

Antioxidant properties of *Helichrysum* endemic species were first analyzed by the TLC-DPPH screening method. The TLC plate was spotted with the crude extract, developed and dried. The TLC plate was then sprayed with a DPPH methanolic solution (0.2%) and incubated in the dark during 30 min. After this period, the active components appeared as yellow spots against a purple background. All extracts of *Helichrysum* showed at least one yellow spot indicating the presence of antioxidant components.

The spectrophotometric DPPH assay results are usually expressed as the efficient concentration (EC_50_) that corresponds to the amount of antioxidant necessary to decrease by 50% the initial DPPH radical concentration. However, this calculation is dependent on the specific conditions used in the assay, chiefly the initial DPPH concentration. Therefore, the construction of a calibration curve of a strong standard antioxidant compound like Trolox or ascorbic acid allows for the interpolation of the values of absorbance variation and the results are expressed as equivalent concentration.

The results obtained for DPPH assay are given in Table 1. A large range of activity was detected, from 23.78 ± 0.75 μmol eq. Trolox 100 g^−1^ (*H. obconicum*, ethyl acetate) to 812.95 ± 1.65 μmol eq. Trolox 100 g^−1^ (*H. melaleucum*, methanol).

With the exception of the *n*-hexane extract, the *H. devium* extracts did not show significant difference (*p* < 0.05).

*H. melaleucum* and *H. obconicum* showed a similar order of activity concerning the solvent polarity, *i.e.*, methanolic extracts gave the highest value followed by *n*-hexane, chloroform and ethyl acetate. Nevertheless, extracts with the solvent of extraction in common gave significantly different (*p* > 0.05) values.

#### 3.5.2. ABTS^•+^ Radical Scavenging Activity

The ABTS assay is based on the neutralization of a radical cation, ABTS^•+^, formed by a single-electron oxidation process [34]. In the presence of electron/hydrogen donors, the ABTS solution turns colorless and this reaction can be accompanied by measuring the decrease in absorbance over time.

As observed in the DPPH method, for *H. devium* samples the *n-*hexane extract presented the highest value of activity of decoloration of ABTS solution (116.4 ± 0.434 μmol eq. Trolox 100 g^−1^) (Table 1).

In general, the values obtained in the ABTS assay are lower than those obtained for the same sample by the DPPH method. This fact can possibly be explained by the presence of compounds with maximum absorptions wavelengths near the working wavelength in the ABTS method (λ = 734 nm).

The presence of tannins, which can react with ABTS better than with DPPH [35] could be a plausible explanation. However, this hypothesis is excluded based on our recent studies [13,17,18] where the polar extracts of *H. devium*, *melaleucum* and *obconicum* were analyzed by HPLC-DAD-ESI/MS^n^ and the major compounds identified were hydroxycinnamic acids, mainly quinic acid derivatives, and flavonoids normally in their *O*-glycosilated form, and no high molecular weight compounds were discovered.

#### 3.5.3. Ferric Reducing Activity (FRAP Assay)

FRAP assay measures the antioxidant capacity of a sample based on its reducing capacity.

The reducing capacity of *Helichrysum* samples varied markedly, with values between 354.8 ± 1.35 mmol Fe (II) mg^−1^ (*H. melaleucum*, hexane) to 19,918 ± 75.4 mmol Fe (II) mg^−1^ (*H. obconicum*, methanol) (Table 1).

For the three species, the highest reducing capacity values were obtained for the methanolic extracts, although with significantly difference between them (*p* < 0.05).

#### 3.5.4. β-Carotene (BC) Bleaching Assay

The evaluation of the antioxidant activity by the β-Carotene assay is based on the fact that the free radical linoleic acid attacks the highly unsaturated β-Carotene; the presence of compounds with antioxidant properties delays the β-Carotene oxidation by neutralizing the free radicals in the medium [22].

In order to compare the percentage of antioxidant activity (AA) of each sample, the same sample concentration was used (1 mg·mL^−1^). At this concentration, none of the samples fully inhibited the β-Carotene oxidation. Nevertheless, all samples prevented, to some extent, the β-Carotene complete oxidation. The registered AA values varied from 22.85% ± 0.29% (*H. melaleucum*, methanol) to 62.82% ± 1.58% (*H. devium*, *n-*hexane) (Table 1).

The methanolic extracts, in general, were less effective in contrast to the results obtained by the other methods, whereas they showed great ability in radical scavenging or reducing properties.

From the three *Helichrysum* species, *H. devium* was the one causing the highest inhibition of β-Carotene oxidation and *H. obconicum* exhibited the lowest capacity to prevent β-Carotene bleaching.

The fact that this method is carried out in emulsion makes its matrix closer to that of real food, but the obtained data are dependent on the polarity of the compounds and, consequently, on their partition between the two phases (aqueous and lipidic). Global results are more sensitive to specific individual components than the other assays. The extracts with a higher AA value contain compounds with high capacity of preventing the oxidation of lipids and could be used as preservatives to delay or limit lipid oxidation.

#### 3.5.5. Correlations between Antioxidant Assays

Table 2a shows the Pearson correlations between TPC and DPPH, ABTS, FRAP and β-Carotene data obtained for *H.* *devium* and *H. melaleucum*. For *H. obconicum,* these correlations were not established since only two points were measured (*n-*hexane extract did not showed any activity and the yield of chloroform extraction was very low to establish its antioxidant activity).

The correlations between TPC and antioxidant assays obtained for *H. melaleucum* extracts were good (*R*^2^ > 0.867). These results are in good agreement with the fact that samples with high levels of phenolic compounds possess, normally, high antioxidant capacity.

In contrast, *H. devium* samples showed a very poor correlation between TPC concerning the DPPH and ABTS assay (*R*^2^ < 0.140). This information suggests that the large percentage of compounds responsible for the antioxidant activity of *H. devium* does not belong to the phenolic type. However, *H. devium* extracts gave a very good correlation between the TPC and the FRAP and β-Carotene results. This behavior was expected due to the fact that the Folin–Ciocalteu method has a mechanism based on the reducing properties of phenolic compounds. It is also possible to infer that the phenolic compounds responsible for the high antiradical scavenging capacity of *H. devium* do not possess an effective reducing capacity.

The correlation between the TPC and β-Carotene values was very good for both plants. These results indicate a great influence of the phenolic components of these two samples in the inhibition of β-Carotene oxidation.

The data obtained by the four methods of antioxidant assays were also correlated using Pearson’s correlation shown in Table 2b.

An excellent correlation between the DPPH and ABTS values was found for *H. melaleucum* (*R*^2^ = 0.997), confirming the same type of reaction involved in the antioxidant measurement. However, for *H. devium,* the correlation was weaker with a *R*^2^ = 0.755. A weak correlation between two mechanistically similar methods, such as ABTS and DPPH, is mainly related to the composition of the sample. Therefore, it is possible to infer that *H. devium* has within its composition compounds with different abilities for scavenging DPPH and ABTS.

**Table 2:** (**a**) The Pearson correlation coefficients between the total phenolic content (TPC) and the antioxidant assays data (DPPH, ABTS, FRAP and BC); (**b**) the Pearson’s correlation coefficients between antioxidant assays data.

(**a**)	***R*^2^**	**DPPH**			**ABTS**	**FRAP**	**BC**
	*H. devium*	0.140			0.054	0.952	0.980
	*H. melalecum*	0.995			0.983	0.867	0.970
(**b**)	***R*^2^**	**DPPH**			**ABTS**		**FRAP**
		*ABTS*	*FRAP*	*BC*	*FRAP*	*BC*	*BC*
	*H. devium*	0.755	0.380	0.036	0.891	0.068	−0.984
	*H. melaleucum*	0.997	0.914	0.990	0.994	0.998	0.926

The FRAP values showed good correlations against DPPH and ABTS for *H. melaleucum* (*R*^2^ > 0.914). A weak correlation between the DPPH and FRAP data for *H. devium* (*R*^2^ < 0.38) was obtained. This fact is linked to the behavior of the *n*-hexane extract in the FRAP method that is opposite to what was observed for the DPPH and ABTS assays where the *n*-hexane extract displayed an antioxidant activity at the same level of the methanol extract. Once more, the reducing properties of the components present in the *H. devium* *n*-hexane extract are evidenced.

The correlations between BC and DPPH/ABTS results were very good for *H. melaleucum* and no correlation was found in *H. devium* sample. In contrast, the FRAP results correlated very well with those of the BC method for *H. devium* and *H. melaleucum.*

### 3.6. Principal Component Analysis (PCA)

PCA was applied to the data set of antioxidant capacity methods, TPC, TFC and *Artemia salina* assays extracts after standardization (the mean of the values for each variable was subtracted from each variable value and the result was divided by the standard deviation of the values for each variable). After standardization, each parameter contributed equally to the data set variance and carried equal weight in principal component calculation.

The loadings, eigenvalues, percentage of cumulative variance (*R*^2^) and cumulative predictive variance (Q^2^) are shown in Table 3. The high dimensionality of the data was reduced to two principal components (PC1 and PC2), totaling 98% of the variance and a very good *R*^2^ (cum) was achieved (0.945), in addition to a good Q^2^ (cum) of (0.397).

PC1 is the component that best approximates the data in the least square sense.

**Figure 2:**
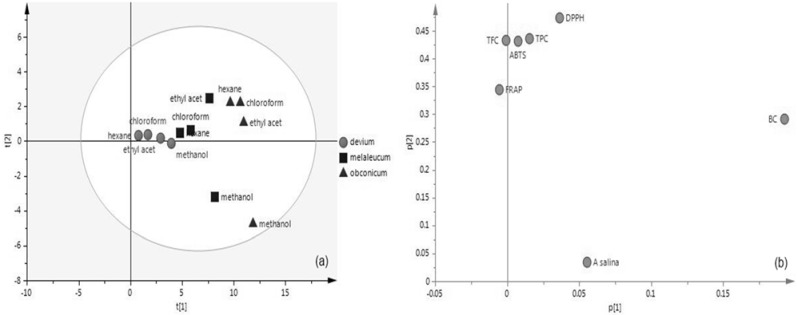
PCA plot of the scores (**a**) and loadings (**b**) of the *Helichrysum* samples.

The scores and loading plots of PC1 *vs.* PC2 are shown in Figure 2. The loading plot displays the relationship between all variables. Variables grouped together are positively correlated like the ABTS, TPC, TFC, FRAP and, to some extent, like DPPH, which is also in good agreement with Pearson’s correlation.

*Artemia salina* and BC are located far from the origin and are the variables with major impact on the model. These variables have a higher impact in separating *H. obconicum* and *H. melaleucum* samples.

The PCA plot segregates *H. devium* on the basis of the FRAP, ABTS, TPC and TFC values. The other two plant extracts are also grouped, with the exception of the methanol extracts that present a deviant behavior due to influence of the *A. salina* variable.

**Table 3:** Loadings, eigenvalues and percentage of cumulative variance for the first three principal components of the data from the *Helichrysum* samples.

Variable	PC1	PC2
TPC	0.01524	0.4357
TFC	−0.00098	0.4327
DPPH	0.03629	0.4742
ABTS	0.00767	0.4315
FRAP	−0.00560	0.34415
BC	0.1924	0.29152
*A. salina*	0.1924	0.03366
*R*^2^ (cum)	0.879	0.945
eigenvalue	5.07	1.63
Q^2^ (Cum)	0.214	0.397

## 4. Conclusions

In the present study, three endemic *Helichrysum* plants used in diet and folk medicine of Madeira Archipelago were analyzed in terms of their antioxidant capacity, toxicity and antimycobacterial power.

Statistically significant differences were observed among the different samples in the different antioxidant assays. For *H. melaleucum* and *H. obconicum,* the methanolic extracts showed the highest antioxidant capacity, whereas the phenolic and flavonoid compounds played an important role in the antioxidant capacity (evidenced by an excellent correlation). For *H. devium,* it is possible to conclude that phenolic compounds are not the only ones responsible for the antioxidant properties since the *n-*hexane extract gave similar or even slightly higher antioxidant values than the methanolic extract.

The PCA analysis allowed the grouping of *H. devium* samples based on the TPC, TFC, ABTS and FRAP variables.

*H. devium* was revealed to be toxic towards *Artemia salina* larvae and to *Mycobacterium tuberculosis*. Further studies with these plant extracts to identify the bioactive compounds and further *in vivo* studies of inhibition are therefore warranted.
